# The first complete mitochondrial genome of the agricultural pest *Micromelalopha sieversi* (Staudinger, 1892) (Lepidoptera: Notodontidae)

**DOI:** 10.1080/23802359.2023.2301005

**Published:** 2024-01-08

**Authors:** Xuan Dai, Qi Chen, Wei Wang, Xing Wang

**Affiliations:** a College of Plant Protection, Hunan Provincial Key Laboratory for Biology and Control of Plant Diseases and Insect Pests, Hunan Agricultural University, Changsha, China; b Research Center for Wild Animal and Plant Resource Protection and Utilization, Qiongtai Normal University, Haikou, China

**Keywords:** *Micromelalopha sieversi*, mitochondrial genome, phylogenetic analysis, Illumina sequencing

## Abstract

*Micromelalopha sieversi* (Staudinger, 1892) is a significant pest of Poplar trees in China. In this study, we used high-throughput sequencing to sequence the whole mitochondrial genome of *M. sieversi*. The length of the genome was 15,373 base pairs. The nucleotide composition was 39.8%, 11.5%, 8.0%, and 40.7% for A, C, G, and T, respectively. We used the maximum-likelihood method to construct a molecular phylogenetic tree based on complete mitogenome sequences of 19 *Noctuoidea* species as ingroups and five *Geometroidea* species as outgroups. The results indicate that the genus *Micromelalopha* is closely related to the genus *Clostera* in family Notodontidae.

## Introduction

Notodontidae is a family of Lepidopteran origin, distributed worldwide with more than 2800 described species, mostly occurring in tropical regions. Moths in this family are medium-sized and adults do not feed. The caterpillars are known for their bizarre appearances and are arboreal insects that cause defoliation of their hosts (Choi et al. 2020). In China, *Micromelalopha sieversi* Staudinger, 1892 (Lepidoptera: Notodontidae) is an important pest of Poplar trees (*Populus* spp., family Salicaceae). Commonly, *M. sieversi* occurs in 3–4 generations in northeast China, but in 5–7 generations in central and southern China. Extensive overlap was found between generations and females did not show significant circadian rhythm-related vocalization behaviors during the light phase but did so during the dark phase (Fan et al. 2015). After mating, females deposit their eggs on the Poplar leaves. The larvae have five instar stages, and the mature larvae pupate in the deciduous layer during winter (Chen et al. 2014), typically causing mesophyll injuries that result in balding of Poplar branches, weakening the host and curtailing growth (Fan et al. 2014; Guo, Liu, Wang, et al. 2019). However, for such a significant pest, most studies have mainly focused on discussing plant-lepidopteran interactions (Bertea et al. 2019; Guo, Liu, Zhang, et al. 2019; Hilker et al. 2023), describing the effects of oviposition behavior on host plants (Guo et al. 2020), and investigating components of sex pheromone (Liu et al. 2019), with little exploration of its phylogeny. The complete mitochondrial genome of this species has not been reported in NCBI and other databases. Therefore, in the current study, we sequenced and annotated for the first time the complete mitochondrial genome (mitogenome) of *M. sieversi* and constructed a molecular phylogenetic tree. This research expands our understanding of its mitogenome characteristics and helps infer the phylogenetic position of the genus *Micromelalopha*.

## Materials

**Figure 1.**
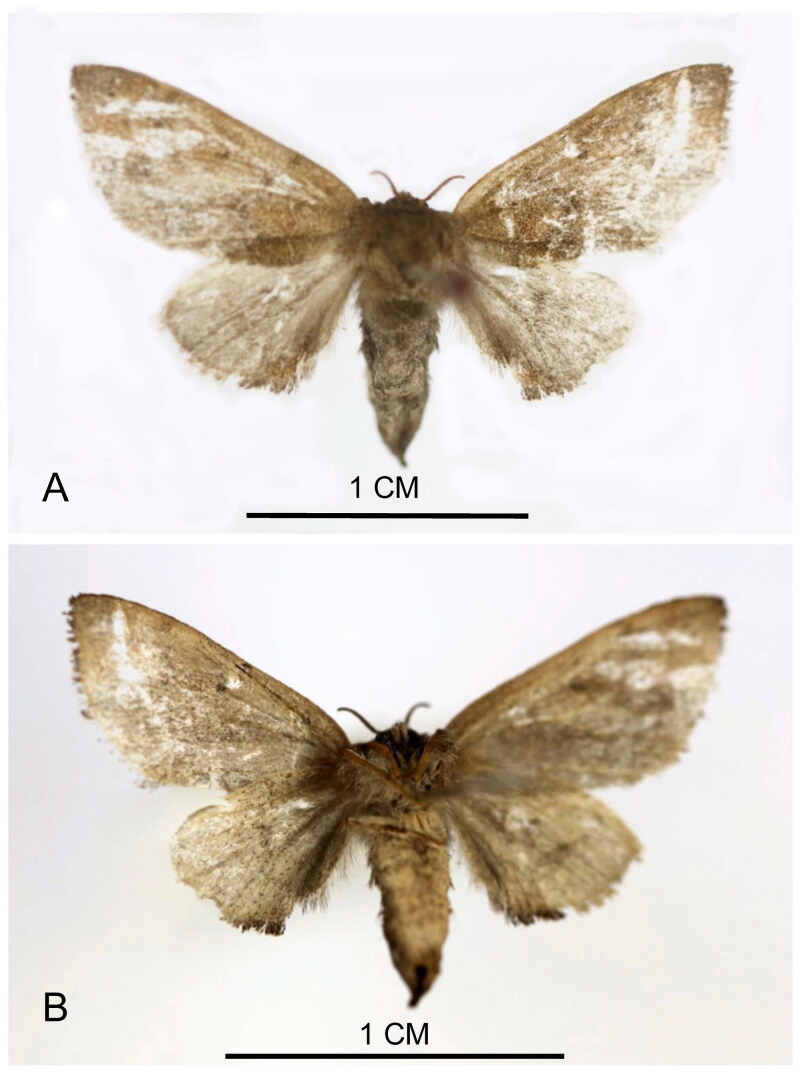
Reference image of adult of *Micromelalopha sieversi*. (A) The dorsal view of *M sieversi*; (B) the ventral view of *M. sieversi*. These images from our specimen were taken by DX.

The specimens of *M. sieversi* (Figure 1) were collected by light trapping on 30 September 2022 at Hanshou National Vegetable Base (28.91°N, 111.95°E, Hanshou, Changde City, China) by Xuan Dai. Meanwhile, the specimens and the genomic DNA were deposited in the Insect Collection of Hunan Agricultural University (contact person: Guo-Hua Huang, email: ghhuang@hunau.edu.cn), Changsha City, Hunan Province, China under the voucher number HAUHL081471.

## Methods

Genomic DNA was extracted from the leg tissue of a single adult using the SteadyPure Universal Genomic DNA Extraction Kit Ver.1.0 protocol and subjected to paired-end sequencing (2 × 150 bp) using an Illumina NovaSeq 6000 platform at Berry Genomics (Beijing, China). The raw reads were filtered using FASTP v0.23.2 (Chen et al. 2018). The genome was assembled *de novo* with NOVOPlasty v4.3.1 (Dierckxsens et al. 2017) and GetOrganelle v1.7.6 (Jin et al. 2020). The *M. sieversi* mitogenome was initially annotated using MITOS web server (Bernt et al. 2013). All tRNA gene structures were predicted and inferred by tRNA scan-SE (Lowe and Chan 2016), with manual adjustments performed using Geneious v.10.1.4 (Kearse et al. 2012). The circular genome map was generated with Circos (Krzywinski et al. 2009), module of MitoZ v3.6 (Meng et al. 2019). To investigate its taxonomic status, we downloaded 19 mitogenomes of Notodontidae species and five outgroup species from the NCBI database for phylogenetic analysis. We manually extracted the amino acid sequences of 13 PCGs in their mitogenomes using Geneious, and aligned them with MAFFT v7.149 (Katoh and Standley 2013). These sequences were then concatenated using FASConCAT-g v1.05.1 (Kück and Longo 2014). Next, we inferred the best partitioning strategy and substitution models using PartitionFinder 2.1.1 (Lanfear et al. 2017) for the concatenated dataset. Finally, we performed a maximum likelihood phylogenetic analysis with five *Geometroidea* species as outgroups using IQtree 2 (Minh et al. 2020). The resulting molecular phylogenetic tree is shown using FigTree 1.4.4.

## Results

**Figure 2.**
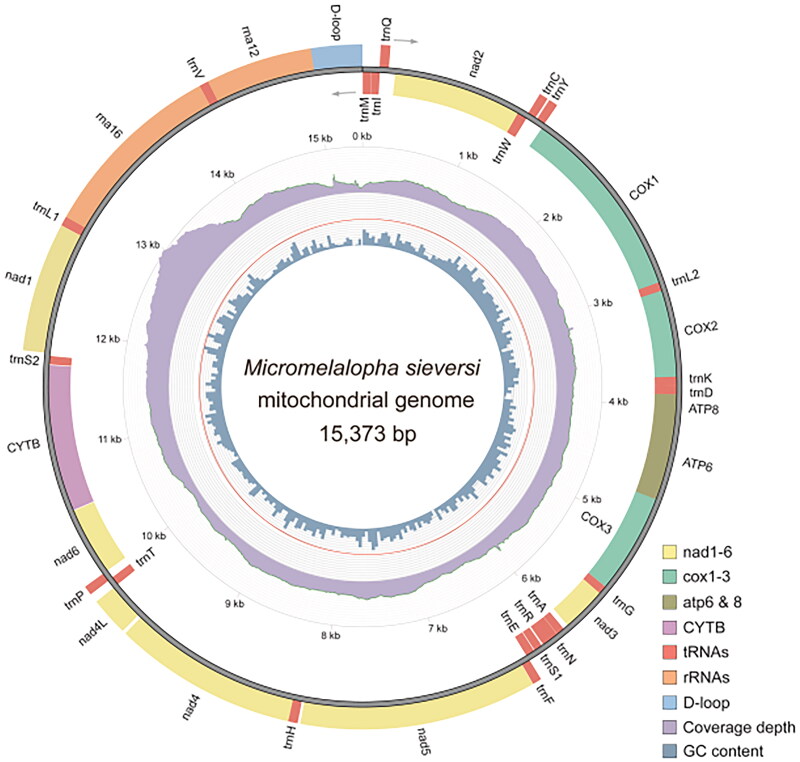
Mitogenome pattern map of *Micromelalopha sieversi*. Grey arrows indicate the direction of gene transcription. Genes inside the circle are coded in the majority strand (J-strand); genes outside the circle are coded in the minority strand (N-strand).

**Figure 3.**
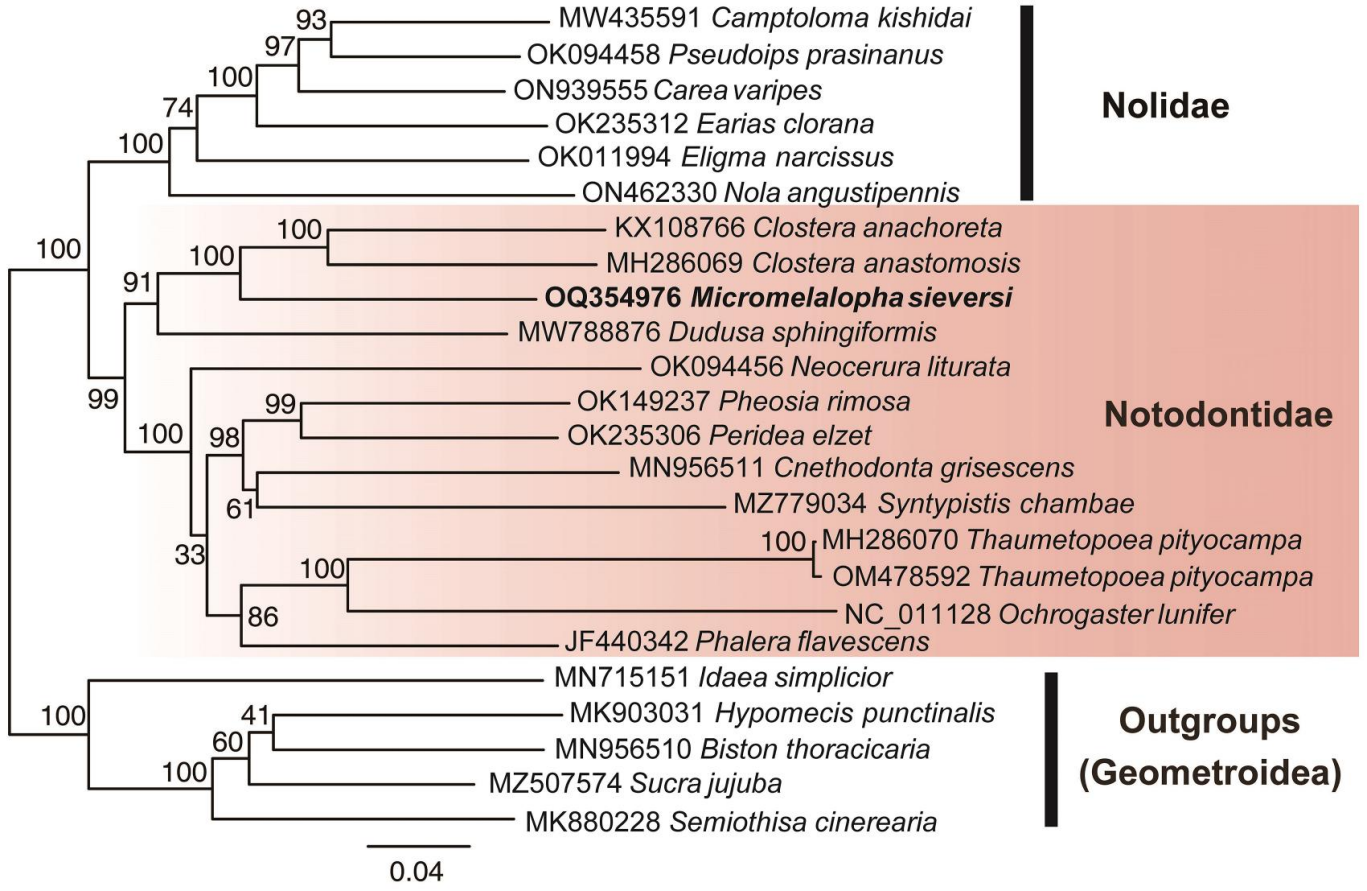
Maximum-likelihood tree of 19 species within the superfamily *Noctuoidea* based on 13 protein-coding genes of mitogenome using five *Geometroidea* species as outgroups. The numbers on the branches indicate ML bootstrap percentages. The following sequences were used: MW435591 *Camptoloma kishidai* (Yuan and Du 2022); OK094458 *Pseudoips prasinanus* (Liang et al. 2022a); ON939555 *Carea varipes* (Liang et al. 2022b); OK235312 *Earias clorana* (Liang et al. 2022c); OK011994 *Eligma narcissus* (Dai et al. 2016); ON462330 *Nola angustipennis* (Hu et al. 2022); KX108766 *Clostera anachoreta* (Zhu et al. 2017); MH286069 *Clostera anastomosis* (Zhu et al. 2018); MW788876 *Dudusa sphigiformis* (Zhou et al. 2021); OK094456 *Neocerura liturata* (Liang et al. 2022d); OK149237 *Pheosia rimosa* (Liang et al. 2022e); OK235306 *Peridea elzet* (Liang et al. 2022f); MN956511 *Cnethodonta grisescens* (Song et al. 2021); MZ779034 *Syntypistis chambae* (Liang et al. 2022g); MH286070 *Thaumetopoea pityocampa*, OM478592 *Thaumetopoea pityocampa* (Wu et al. 2019); NC_011128 *Ochrogaster lunifer* (Salvato et al. 2008); JF440342 *Phalera flavescens* (Sun et al. 2012); MN715151 *Idaea simplicior* (Song et al. 2019); MK903031 *Hypomecis punctinalis* (Sun et al. 2021); MN956510 *Biston thoracicaria* (Huang et al. 2021); MZ507574 *Sucra jujuba* (Liu et al. 2014); MK880228 *Semiothisa cinerearia* (Chi et al. 2022).

The full mitogenome of *M. sieversi* (GenBank accession number: OQ354976) is 15,373 bp in size. The mitogenome has an AT-rich region along with 13 protein-coding genes (PCGs), 22 transfer RNA (tRNA) genes, and two ribosomal RNA (rRNA) genes. Among these, 15 genes are transcribed on the minority strand (N strand), and the remaining 22 genes are transcribed on the majority strand (J strand). The nucleotides consist of A, C, G, and T, accounting for 39.8%, 11.5%, 8.0%, and 40.7%, respectively. The AT nucleotide content is 80.5% (Figure 2). The nucleotide length among the genes is 227 bp, distributed among 18 pairs of adjacent genes, with lengths ranging from 2 to 53 bp. There are 35 bp overlapping nucleotides, scattered between 12 pairs of adjacent genes, with length ranging from 1 to 9 bp. The length of A + T rich region between 12S rRNA and trnM is 373 bp. The molecular phylogenetic tree based on complete mitogenome sequences is given in Figure 3. The family Notodontidae has polyphyletic groups, with one clade being sister to the association of Nolidae + (Erebidae + Noctuidae) with very weak support (41%) and the other clade being sister to all other *Noctuoidea* with strong support (100%). The genus *Micromelalopha* was closely related to the genus *Clostera* in the family Notodontidae that was a clade strongly supported by the bootstrap value of 100%.

## Discussion and conclusions

As the first report on the mitogenome of the genus *Micromelalopha*, its gene order and AT content are highly similar to other Notodontidae mitogenomes (Zhu et al. 2017, 2018). Phylogenetic inference based on the 13 PCGs of this mitogenome shows a relatively clear positioning of the genus *Micromelalopha* within Notodontidae. Taken together, the mitogenome of *M. sieversi* could contribute to our understanding of mitogenome characteristics and help infer the phylogenetic position of the family Notodontidae.

## Supplementary Material

Supplemental Material

## Data Availability

The genome sequence data that support the findings of this study are openly available in GenBank of NCBI at under the accession no. OQ354976. The associated BioProject, BioSample, and SRA numbers are PRJNA929425, SAMN32958594, and SRR23269617, respectively.
