# Supplementary figures and images for: The first complete mitochondrial genome of the agricultural pest Micromelalopha sieversi (Staudinger, 1892) (Lepidoptera: Notodontidae)
Source: Mitochondrial DNA B Resour. 2024 Jan 8;9(1):50–4. doi: 10.1080/23802359.2023.2301005 (PMC10776056; doi:10.1080/23802359.2023.2301005)

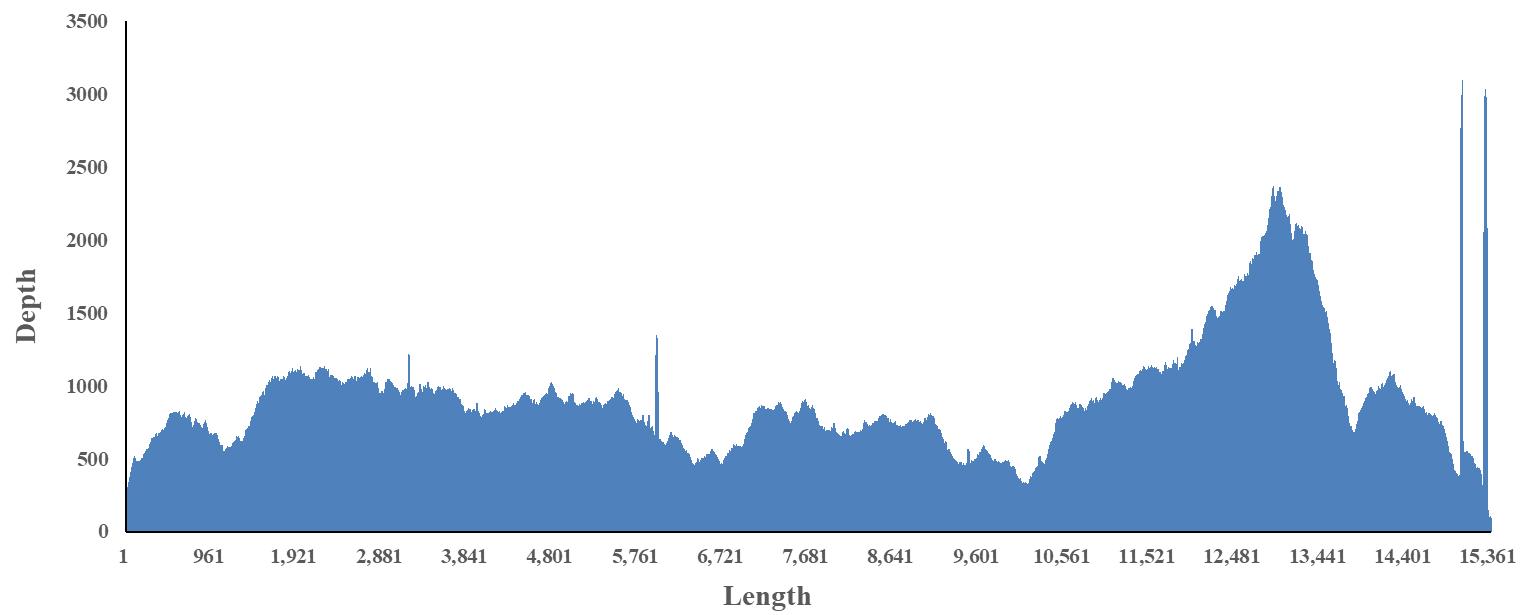

Supplement: Supplemental Material [file TMDN_A_2301005_SM0784.jpg]
